# The Joint Action of Destruxins and Botanical Insecticides (Rotenone, Azadirachtin and Paeonolum) Against the Cotton Aphid, *Aphis gossypii* Glover

**DOI:** 10.3390/molecules17067533

**Published:** 2012-06-18

**Authors:** Fei Yi, Chunhua Zou, Qiongbo Hu, Meiying Hu

**Affiliations:** College of Resources and Environment, South China Agricultural University, Guangzhou 510602, China

**Keywords:** destruxins, rotenone, paeonulum, azadirachtin, *Aphis gossypii*

## Abstract

The joint action of destruxins and three botanical insecticides, rotenone (Rot), azadirachtin (Aza) and paeonolum (Pae) against the cotton aphid, *Aphis gossypii*, was bioassayed. In laboratory experiment, several synergistic groups of destruxins with botanical insecticides were found by means of Sun’s Co-toxicity Coefficients (CTC) and Finney’s Synergistic Coefficient (SC). The best synergistic effect was discovered in the ratio group Des/Rot 1/9 with the CTC or SC and LC_50_ values of 479.93 or 4.8 and 0.06 μg/mL, respectively. The second and third synergistic effects were recorded in the ratio groups Des/Rot 7/3 and 9/1. Although the ratio groups Des/Aza 6/4, Des/Pae 4/6, 3/7 and 2/8 indicated synergism by Sun’s CTC, they were determined as additive actions by Finney’s SC. Additive actions were also found in most of the ratio groups, but antagonism were recorded only in three ratio groups: Des/Pae 9/1, 7/3 and 6/4. In greenhouse tests, the highest mortality was 98.9% with the treatment Des/Rot 1/9 at 0.60 μg/mL, meanwhile, the treatments Des/Pae 4/6 and Des/Aza 6/4 had approximately 88% mortality.

## 1. Introduction

The cotton aphid, *Aphis gossypii* Glover, is a destructive pest of over 24 different crops worldwide. It not only feeds directly on crops, but also causes serious damage to plants through its role as a vector of viruses [1]. The application of chemical insecticides has long been the most important method of controlling this pest; however, the species has now developed resistance to many available pesticides; in addition, there is growing global concern over the environmental impacts of pesticide use. In China, *A. gossypii* has become resistant to fenvalerate (chemical class: pyrethroid), imidacloprid and acetamiprid (neonicotines) and omethoate (organophosphates), among others [2]. In Europe, strong resistance has been found to pirimicarb (carbamate) and oxydemeton-methyl (organophosphates) and, to a lesser extent, to cyfluthrin (pyrethroid) [3]. Therefore, there is an essential and urgent need to develop new pesticides, or new combinations of existing compounds, to gain effective control of this pest.

Destruxins are a type of mycotoxin extracted from species of entomopathogenic fungi, such as *Metarhizium anisopliae* and *Aschersonia* spp. [4]. They have the molecular structure of a cyclic hexadepsipeptide and are typically composed of five amino acids and an α-hydroxy acid; in addition, 35 destruxin analogs have been reported [5,6]. The insecticidal activities of destruxins against aphids and other pests have been described in several reports [7,8,9,10]. In addition, destruxins also have synergism with Bt, saponins and entomopathogenic fungi [11]. Although the molecular mechanisms behind the lethal effect of destruxins on insects are not yet clear, destruxins are known to inhibit the insect immune system; this distinguishes them from other available pesticides and suggests the possibility of a new class of insecticide [12]. However, destruxins have not yet been applied under field conditions; therefore, exploring their interactions with other insecticides, including botanical insecticides, will enhance knowledge of their insecticidal effects.

Many natural botanical insecticides, including azadirachtin and rotenone, have attracted research interest because of their lower toxicity to mammals, less environmental residue, inability of pests to develop resistance against them, and their multiple activities and modes of action [13]. The most famous botanical insecticide, azadirachtin, is a type of limonoid extracted from the neem tree *Azadirachta indica*. It has various insecticidal activities, including killing, deterring, antifeeding and inhibiting the growth of more than 250 species of agricultural, forestry, medical and veterinary pests, including aphids [14,15,16]. In addition, azadirachtin is known to act synergistically with other chemical and biological pesticides [17,18]. In China, there are 16 registered uses of azadirachtin and its mixtures for controlling various insect pests, including the diamond-back moth (*Plutella xylostella*), cotton leaf worm (*Spodoptera litura*) and other vegetable and fruit crop pests [19].

Rotenone, another important botanical insecticide extracted from various plants, including species of *Derri*s, *Millettia, Tephrosia* and *Lonchocarpus*, has been shown to be an effective control agent of many pest species, including aphids, mites and moths [20,21,22,23]. In China, there are 19 rotenone-based insecticides licensed to control aphids, the diamondback moth and other important pests of vegetables [24].

By contrast, paeonol, a phenyl compound from *Cynanchum paniculatum*, *Paeonia suffruticosa* and *Paeonia lactiflora*, is usually used as a Chinese medicine; however, there have been a few reports about its possible use as an insecticide and acaricide [25,26].

## 2. Results

### 2.1. The Joint Action of Destruxins and Rotenone

In total, synergistic or additive interactions were recorded for all ratio groups of destruxin mixed with rotenone (Table 1). The best synergistic effect was found in the ratio group Des/Rot 1/9 for its CTC or SC values of 479.93 or 4.8 with a LC_50_ of 0.06 μg/mL. Similarly synergistic actions were recorded in the ratio groups Des/Rot 7/3 and 9/1 with CTC values of 427.06 and 374.40 or SC values of 4.3 and 3.7, respectively. The other treatment ratio groups were determined as additive effects according to their CTC and SC values (Table 1).

**Table 1:** The LC-p equations, LC_50_s, CTCs and SCs of destruxins and rotenone (alone and in combination) against cotton aphids.

* Ratio group	Intercept	Slope	R	χ^2^	p	LC_50_ (μg/mL, 24 h) (95%CI)	CTC	SC
10/0	3.3461	1.74103	0.9933	0.2610	0.9672	8.91 (3.94–13.56)	–	
9/1	5.2003	0.7679	0.9957	0.1449	0.9860	0.55 (0.24–0.88)	374.40	3.7
8/2	4.8969	0.8421	0.9988	0.0515	0.9969	1.33 (0.72–2.01)	87.53	0.9
7/3	5.7584	1.0724	0.9994	0.0380	0.9981	0.19 (0.13–0.28)	427.06	4.3
6/4	5.0343	1.6447	0.9911	0.9108	0.8228	0.95 (0.69–1.22)	65.55	0.7
5/5	5.2913	1.0904	0.9980	0.1392	0.9867	0.54 (0.37–0.77)	93.57	0.9
4/6	5.4039	1.2696	0.9904	0.8257	0.8433	0.48 (0.36–0.67)	88.56	0.9
3/7	5.4169	1.0221	0.9961	0.2255	0.9734	0.39 (0.26–0.57)	94.06	0.9
2/8	5.5559	1.0899	0.9942	0.4190	0.9363	0.31 (0.19–0.43)	104.08	1.0
1/9	6.2325	0.9993	0.9932	0.3516	0.9501	0.06 (0.03–0.09)	479.93	4.8
0/10	5.7492	1.2937	0.9852	0.8893	0.8280	0.26 (0.17–0.37)	–	

* The ratio of destruxins/rotenone.

### 2.2. The Joint Action of Destruxins and Azadirachtin

The mixtures of destruxins and azadirachtin showed different results depending on their ratios (Table 2). In accordance with Sun’s method, the ratio group Des/Aza 6/4 was determined as synergistic for its CTC value of 191.08, but antagonistic effects were found in the ratio groups Des/Aza 8/2 and 1/9 for their CTC < 50, then, additive effects were recorded in other ratio groups. However, based on Finney’s standard, the treatment Des/Aza 6/4 was determined as an additive effect for its SC value >0.4 and <2.7. Additive actions were also found in the other ratio groups as well (Table 2).

**Table 2:** The LC-p equations, LC_50_s, CTCs and SCs of destruxins and azadirachtin (alone and in combination) against cotton aphids.

* Ratio group	Intercept	Slope	R	χ^2^	p	LC_50_ (μg/mL, 24 h) (95%CI)	CTC	SC
10/0	3.3461	1.74103	0.9933	0.2610	0.9672	8.91 (3.94–13.56)	–	
9/1	4.0932	1.0829	0.9672	2.2372	0.5247	6.88 (5.03–10.26)	136.96	1.4
8/2	3.4689	1.1566	0.9962	0.2228	0.9738	21.08 (14.53–31.07)	47.43	0.5
7/3	3.7462	1.0091	0.9959	0.2460	0.9698	17.48 (11.66–25.14)	60.91	0.6
6/4	4.4559	0.7019	0.9959	0.0612	0.9961	5.96 (1.32–12.97)	191.08	1.9
5/5	3.7304	0.9889	0.9992	0.0462	0.9974	19.23 (12.49–29.12)	63.65	0.6
4/6	3.6227	1.0338	0.9973	0.1460	0.9858	21.49 (13.77–53.71)	61.55	0.6
3/7	3.8359	0.8564	0.9317	2.9930	0.3927	22.87 (12.91–83.67)	62.92	0.6
2/8	3.6663	1.0436	0.9936	0.3817	0.9440	18.97 (12.93–27.35)	83.16	0.8
1/9	3.1541	1.1625	0.9954	0.3372	0.9529	38.71 (27.17–54.67)	45.10	0.5
0/10	3.5996	1.0847	0.9981	0.1050	0.9912	19.54 (13.59–34.93)	–	

* The ratio of destruxins/azadirachtin.

### 2.3. The Joint Action of Destruxins and Paeonolum

There were different joint actions between destruxins and paeonolum. According to Sun’s standard, synergistic effects were found in the ratio groups Des/Pae 4/6, 3/7 and 2/8 according to their CTC values of 200-250, but antagonistic effects were recorded in the groups Des/Pae 9/1, 7/3 and 6/4 for their CTCs < 50, meanwhile, additive effects were found in other groups. However, based on the Finney’s standard, although similarly antagonistic effects were found in the groups Des/Pae 9/1, 7/3 and 6/4, additive effects and no synergisms were recorded in any other group (Table 3).

**Table 3:** The LC-p equations, LC_50_s, CTCs and SCs of destruxins and paeonolum (alone and in combination) against cotton aphids.

* Ratio group	Intercept	Slope	R	χ^2^	p	LC_50_ (μg/mL, 24 h) (95%CI)	CTC	SC
10/0	3.3461	1.7410	0.9933	0.2610	0.9672	8.91 (3.94–13.56)	–	
9/1	3.8739	0.8644	0.9857	0.9298	0.8182	20.08 (14.88–29.67)	43.71	0.4
8/2	4.2234	0.8429	0.9839	1.0993	0.7772	8.34 (6.06–11.58)	103.70	1.0
7/3	3.6315	1.0200	0.9978	0.1233	0.9889	21.96 (15.13–32.88)	38.81	0.4
6/4	3.9967	0.7652	0.9862	0.6620	0.8821	20.47 (12.99–47.44)	41.05	0.4
5/5	4.0469	0.8405	0.9947	0.2683	0.9659	13.61 (9.11–27.01)	60.87	0.6
4/6	4.3904	1.1657	0.9931	0.6012	0.8962	3.33 (2.24–4.43)	245.32	2.5
3/7	4.5016	0.9585	0.9961	0.2904	0.9618	3.31 (2.30–4.56)	243.43	2.4
2/8	4.3131	1.1654	0.9871	1.5967	0.6601	3.89 (3.04–4.95)	204.34	2.0
1/9	4.2580	0.9500	0.9968	0.2251	0.9735	6.04 (4.29–10.24)	129.85	1.3
0/10	3.5990	1.5765	0.9820	4.4224	0.2193	7.74 (6.49–9.20)	–	

* The ratio of destruxins/paeonolum.

### 2.4. Greenhouse Experiments

In the greenhouse experiments, there were significant differences among the treatments (Table 4). Mortality percents >96% were found in three treatments: Des/Rot 1/9 (Des = 0.06, Rot = 0.54, total = 0.60 μg/mL), Des/Mat 3/7 (Des = 0.18, Matrine = 0.42, total = 0.60 μg/mL) and Mat (matrine), which were significantly (*P *< 0.05) higher than the mortality percents of other treatments, except the Rot treatment with 91.6%. However, in contrast to other treatments with higher total insecticide concentrations, the treatment Des/Rot 1/9 and Des/Mat 3/7 with the lowest total concentration of 0.60 μg/mL achieved significantly higher control effects.

**Table 4:** Results from the greenhouse experiments.

Treatment	Insecticide and its concentration (μg/mL)	* Mortality (%) (Mean ± SE)
Des/Rot 1/9	Des = 0.06, Rot = 0.54, total = 0.60	98.9 ± 1.2^ A^
Des/Mat 3/7	Des = 0.18, Mat=0.42, total = 0.60	98.9 ± 0.7^ A^
Des/Pae 4/6	Des = 1.60, Pae = 2.40, total = 4.00	88.6 ± 2.3^ B^
Des/Aza 6/4	Des = 36.00, Aza = 24.00, total = 60.00	88.9 ± 1.2^ B^
Mat	Mat = 50.00	96.7 ± 2.9^ A^
Rot	Rot = 3.00	91.6 ± 1.1^ AB^
Des	Des = 100.00	87.8 ± 2.8^ B^
Pae	Pae = 80.00	85.7 ± 1.4^ B^
Aza	Aza = 200.00	85.3 ± 3.0^ B^
Control		1.8 ± 0.3

* The means labeled the same letters indicated that they were insignificant differences at p = 0.01, as tested by a Tukey test.

## 3. Discussion

In this study, different joint actions were discovered for the mixtures of destruxins and botanical insecticides. In general, there were synergistic or additive effects in destruxins mixed with rotenone, azadirachtin or paeonolum, except for a few ratio groups. In particular, when destruxins were mixed with rotenone at a ratio 1/9, an extremely low LC_50_ value of 0.06 mg/mL was recorded. The level of the synergism is more obvious than for the botanical insecticides mixed with chemical or biological insecticides [27,28,29,30], although it was slightly lower than that for destruxins mixed with matrine described by the authors [31]. In the greenhouse trial, best control effects on the aphids were also found in the mixtures of destruxins with rotenone and matrine. These results suggest that the mixing destruxins with each of these botanical insecticides at an appropriate ratio would result in a mixture that might be used to control cotton aphids.

In practice, using the mixture of the various classes of insecticides is an important part of insecticide application to increase the control effects, to reduce the costs and avoid the insects developing resistance against an individual pesticide. A similar strategy is often found in the area of medicine, for example, in the cocktail therapy was used to treat patients with HIV/AIDS [32]. However, the interaction of different insecticides usually has three results: addition, antagonism and synergism. Whether synergism occurs is influenced by many factors, such as the insect species and class of insecticides used. Biochemically, the different penetration, absorption, detoxication and target proteins of insecticides determine their interaction effects, generally, synergistic actions are preferably found in the mixtures of the insecticides targeting on different proteins or organs and tissues [33].

Destruxins and botanical insecticides have different mechanisms of action. It is generally recognized that destruxins target the immune system of the insects, causing damage to the hemocytes [12], suppressing phagocytic activity [34] and the expression of various antimicrobial peptides [35]. Destruxins are also often found to act more slowly on insects [9]. By contrast, rotenone is known to be an inhibitor of the respiratory chain, preventing the transport of electrons from NADH to CoQ. Azadirachtin has the behavior regulation properties of an antifeedant and deterrent for many insects, and it also disrupts insect growth, although it acts slowly [36]. However, the insecticide mechanism of paeonolum is still unknown. As far as the large difference of action mechanisms are concerned, the certain synergistic action between destruxins and the three botanical insecticides can be accepted. Probably, the synergistic action seen between destruxins and rotenone might be related to their different target systems. In addition, the slower pest-killing speed of destruxins in combination with the quicker pest-killing speed of rotenone might also contribute to this synergistic interaction. However, the antifeedant and deterrent actions of azadirachtin do not prevent aphids from feeding on the plants, because aphids suck the phloem juices of host plants. It might be related to the non-synergistic effects of destruxins mixed with azadirachtin. 

Furthermore, the methods for evaluating joint action usually result in different conclusions. In this experiment, we used Sun’ CTC and Finney’s SC standards to determine if synergistic effects existed in the different insecticide ratio groups. We can give different conclusions for an identical treatment, for example, the ratio groups Des/Pae 4/6, 3/7 and 2/8 were determined to have synergistic effects by Sun’s CTC and additive effects by Finney’s SC. It seems that Finney’s SC has a more severe standard than Sun’s CTC to define a synergistic effect, so researchers should select the most suitable methods to evaluate joint action.

In this experiment, we also found that there were large changes in the different ratio groups of destruxins with the three botanical insecticides. The phenomenon was probably related to the impurity of compounds used in the experiment. The 30.4% destruxins contains as active ingredients destruxin A and B, in addition, destruxin E and other destruxin homologues, *etc*., and many other unknown components. Similarly, 34% azadirachtin includes many homologues and other unknown materials. Of course, rotenone and paeonolum are not pure, although their contents are more than 95%. Multiple components of insecticides probably make understanding the interactions more complicated. However, the trends of synergism between destruxins and botanical insecticides found in this experiment should be useful as a reference. Undoubtedly, the more acute interactions and the mechanisms of synergisms between destruxins and the botanical insecticides should be the focus of future research.

## 4. Materials and Methods

### 4.1. Insects

A population of aphids was collected from local fields and moved onto potted taro *Colocasia esculenta *(L.) Schoot plants, which were then placed in a greenhouse. Once the aphids had reproduced for two generations, similar non-winged adults were selected for the bioassay.

### 4.2. Insecticides

The insecticides investigated in this study were as follows: 95% rotenone (Rot) (Sigma-Aldrich, St. Louis, MO, USA); 98% paeonolum (Pae) (Xian Zhongxin Biotechnology Co., Ltd, Xian, China); azadirachtin (Aza) powder (prepared by the authors with reference to Wang *et al*. [37]; the content of azadirachtin was 34% determined by HPLC); 34.2% destruxins (Des) powder (prepared by referring to Hu *et al*. [38]; the content of destruxin A and B were 30.4% and 3.8%, respectively, as determined by HPLC).

### 4.3. Bioassays

Insecticides were dissolved in acetone to a concentration of 100,000 μg/mL, and then diluted with 0.05% Tween 80 into stock solutions at a concentration of 10,000 μg/mL. Working solutions were diluted from stock solutions with 0.05% Tween 80. Serial mixtures were prepared with Des/Rot (or Pae, Aza) at a ratio of 10/0, 9/1, 8/2, 7/3, 6/4, 5/5, 4/6, 3/7, 2/8, 9/1 or 0/10. Controls were 0.05% Tween 80 solutions with acetone at the same concentration as the treatment solutions. A leaf-immersing method was used as the bioassay, Detached leaves (4 cm × 4 cm) were cut from the potted plants and checked under a stereomicroscope to ensure that approximately 20 similar non-winged adult aphids remained on each leaf. The leaves were then dipped into the insecticide solutions and controls for a total of 5 s. After drying, the leaves were put into Petri dishes (diameter 9 cm) with wet cottonwool balls and cultured at temperature 25.0 °C and photoperiod 14:10 h light:dark. Three leaves were used for each treatment and the experiments were duplicated twice [39].

### 4.4. Statistical Analysis

Mortality was scored 24 h after treatment. Aphids were considered dead if their antenna showed no movement in response to a physical stimulus (touch). Data were corrected for control mortality [40] and analyzed by probit analysis [41] using the software DPS [42]. Sun’s Co-toxicity coefficients (CTC) [43] and Finney’s synergistic coefficient (SC) [41] were used to determine the joint action of the solutions: if CTC > 150 or SC > 2.7, it shows a synergistic effect, whereas CTC < 50 or SC < 0.4 indicates an antagonistic effect, other CTCs and SCs give additive effects.

### 4.5. Greenhouse Experiments

In greenhouse experiments, the average temperature was 24.0 °C (18.5–35.5 °C) and the photoperiod was 14:10 h light:dark. Assay units were pot-planted *C. esculenta* approximately 40 cm high with three leaves, on which there were at least 300 non-winged aphids. Based on the results of the laboratory tests, eight treatments were investigated in the greenhouse experiments. In addition, 0.05% Tween 80 was used as a control (Table 4). Insecticide solutions were diluted with 0.05% Tween 80. Each treatment was assigned three potted plants. Each potted plant was sprayed with 30 mL insecticide solution. Before and 24 h after treating, the number of aphids was surveyed and then the number of mortalities was evaluated according to Abbott’s equation. Every treatment and control was replicated three times. The data were analyzed using the F-test and Tukey test employing DPS software [42].
